# Replacing Iodine Staining with Size of Lesion: The Performance of Modified Reid Colposcopic Index

**DOI:** 10.31557/APJCP.2019.20.10.3021

**Published:** 2019

**Authors:** Sathone Boonlikit, Pornrapee Arnont

**Affiliations:** 1 *Division of Gynecologic Oncology, Department of Obstetrics and Gynecology, Rajavithi Hospital, College of Medicine, Rangsit University, Bangkok, *; 2 *Department of Obstetrics and Gynecology, Pathum Thani Hospital, Pathum Thani, Thailand. *

**Keywords:** Colposcopic index, Swede colposcopic score, Colposcopy

## Abstract

**Aim:**

This study of diagnostic accuracy aimed to assess the performance of authors’ proposing colposcopic index for detecting histological diagnosis of cervical intraepithelial neoplasia grade 2 or worse (CIN2 or worse).

**Methods:**

Retrospective analysis of data was carried out on medical records of women who underwent colposcopy in Rajavithi hospital from January 2007 to December 2014. The authors’ proposed score included the first 3 criteria of RCI (margin, color, vascular pattern) and replacing the last RCI criterion (iodine staining) with the detail of size and location of a lesion which was retrospectively retrieved from medical records. Total score for detecting any lesion was ranged from 0-8, similar to the RCI. Performance of the score was assessed for sensitivity, specificity, and positive and negative predictive values at every cut-off level.

**Results:**

Among 207 eligible women, 87 (42%) had CIN2 or worse. Cut-off level of score ≥ 6 had a sensitivity, specificity, and positive and negative predictive values of 54.0%,97.5%,94.0%,74.5%, respectively while cut-off value ≥ 2 had sensitivity , specificity, positive and negative predictive values of 94.2% ,55.8% ,60.7%, and 93.0%, respectively, for histological diagnosis of CIN 2 or worse. The area under ROC curve was 0.88. In women with type 3 T-zone, the area under ROC curve was 0.94 which was excellent.

**Conclusion:**

The performance of the colposcopic score that replaces iodine staining with the size and location of the lesion is good and practical. High cut-off level can be used in see and treat approach for high-grade squamous intraepithelial lesions. Low cut-off level may be used for omitting biopsy in case of low grade impression. This scoring system seems to have greater performance in womens with type 3 T- zone.

## Introduction

Colposcopy is a standard method for diagnosis of cervical intraepithelial neoplasia (CIN). The major purpose of colposcopy is to find the abnormality with the main goal to identify the area of epithelium that has the highest degree of disease and to direct biopsy. Grade of CIN or occult early invasion can be identified and histology can be obtained by colposcopically directed biopsy (CDB). Management algorithms are based on the colposcopic findings and the histology from CDB (Massad et al., 2013). However, there is not perfect correlation between the visual changes of the cervical epithelium and the severity of the preneoplastic and neoplastic changes. The most prominent areas of colposcopic change do not necessarily coincide with areas of greatest histologic severity. Reid and Scalzi (1985) introduced Reid colposcopic index (RCI) which has been commonly used. The RCI takes into account four colposcopic features of premalignant cervical lesions to achieve this predictive accuracy. The four colposcopic criteria used are: (1) the margin of the lesion, (2) the color of the acetowhitening, (3) the type of vascular pattern, and (4) the iodine staining reaction (Reid and Scalzi,1985). The accuracy and correlation of RCI have been reported to be high in some investigations (Reid and Scalzi, 1985; Mousavi et al., 2007; Boonlikit, 2011; Boonlikit, 2016; Ranga et al., 2017). However, the accuracy of colposcopy has been increasingly questioned because some studies reported that accuracy and correlation with histology were imperfect (Massad and Collins, 2003; Jeronimo and Schiffman, 2006; Ferris and Litaker, 2006; Massad et al., 2009). Recent studies raised concerns about current colposcopic practice based on the RCI alone (Ferris and Litaker, 2006; Massad et al., 2009). There have been efforts in modifying RCI to improve accuracy (Strander et al., 2005; Ferris and Litaker, 2006; Hong et al., 2010). Strander, et al., (2005) have designed a modified scoring system which includes lesion size and location as independent variables. Performance of the new score was impressive and correlated well with RCI (Strander et al., 2005; Bowring et al., 2010; Ranga et al., 2017). In our institute, RCI yielded a good performance and correlation with histology from CDB and it had high accuracy in differentiating high-grade lesion (CIN2/3) from low-grade lesion(HPV/CIN1) (Boonlikit, 2011; Boonlikit, 2016). Nevertheless, the individual criteria do not carry equal value. Scoring by iodine staining is considered problematic because it is very inconvenient for the patient and has poor predictive value of high-grade lesion (Reid and Scalzi, 1985; Boonlikit, 2011; Campion and Canfell, 2015). This criterion is preferable used in demonstrating and training. The other disadvantage of RCI is that the score does not include the size of a lesion. From the literature, the size of lesion is strongly predictive of high-grade lesion or occult invasive (Ji et al., 1991; Shafi et al., 1991; Kierkegaard et al., 1995; Sellors and Sankaranarayanan, 2003; Campion and Canfell, 2015). Incorporating size and location of lesion in the existing colposcopic score in the previous studies is potentially useful. The primary aim of this evaluation is to examine the performance of diagnosis of CIN2 or worse using the authors’ proposing colposcopic index that omits the score of iodine staining and replaces it with the size and location of the lesion in the transformation zone. In addition, the authors would like to validate this modified colposcopic index in our institute.

## Materials and Methods

The study was approved by the Institutional Review Board of Rajavithi hospital. Retrospective analysis of data was carried out on medical records of women who underwent colposcopy in Rajavithi hospital from January 2007 to December 2014. In our institute, all women with cytologic abnormalities (ASC-US or worse) underwent colposcopy, a punch biopsy was taken from the worst affected area via colposcopic guidance using 3% acetic acid (CDB). Cases were eligible in the present study if they met the author’s inclusion criteria, which was the women underwent colposcopy with RCI scoring. All eligible cases were collected consecutively. The scoring system for deriving the RCI is detailed in the literature (Reid and Scalzi, 1985; Greenberg, 2002; Sellors and Sankaranarayanan, 2003; Boonlikit, 2011; Campion and Canfell, 2015). Report of histology from CDB taken at the scored area must be available. Pregnant women, women with unavailable histology from CDB, women with history of pelvic radiotherapy were excluded from the study. RCI scoring was performed by one colposcopist who knew the result of the referral smear. All cases were colposcopically scored by 3 criteria as follows: the margin of the lesion, the color of the acetowhitening, the type of vascular pattern. The colposcopic criteria were scored in the 0 point, 1 point, and 2 points. In our institute, Lugol’s iodine staining was not routinely applied and only some women were scored by this criterion. Most of women were scored by the first three criteria. Then, CDB were immediately taken from the highest-score lesion. In some cases, when lesions were large and multiple CDB were done on the same cervix, only one result from the highest-score location of lesion was analyzed. In our colposcopy unit, a detail of size and location of a lesion has been routinely recorded in colposcopy chart in a usual practice. Therefore the data can be retrospectively retrieved without difficulty. Size of the lesion was assessed and recorded by two methods as follows: 1) assessment of number of cervical quadrants which the colposcopically visible lesions covered regardless of the type of cervical transformation zone visualization, 2) measurement of the size lesion was also accomplished by placing small cotton tipped applicators, which was approximately 5 mm in diameter in our clinic close to the lesion and comparing the lesion with the cotton as measuring tool. In case of incomplete visualization of the cervical transformation zone, only part of the lesion colposcopically visible was considered as measurable lesion. 

In cases with inadequate data, the colpophotograph were then reviewed and the detail of size and location of the lesions were assessed and recorded by colposcopists. Scoring in this criterion was adapted from the Swede score, a scoring system proposed by Strander et al., (2005). 

Likewise, the “size and location” criterion was scored in 0 point, 1 point, and 2 points. As a result, total scoring detects for any lesion ranged from 0-8, similar to the total score of RCI. These were detailed in Table 1. Pathologic evaluation of all specimens was performed by 4 pathologists without any detail of RCI. However, they were not blinded for the result of the referral smear and colposcopic impression. Performance of this scoring system was assessed for sensitivity, specificity, and positive and negative predictive values at every cut-off level. The reference standard was the histopathology result from CDB where the lesion was scored because it represented the severity of local histopathological change at that site. The performance of score was also evaluated using the area under the ROC curve. The data were analyzed using the statistical package for the social science (IBM^®^SPSS^®^ for Windows) version 22.0 (IBM Corp., Armonk, NY, USA).Significance was set at the level of α = 0.05.

## Results

**Figure 1:**
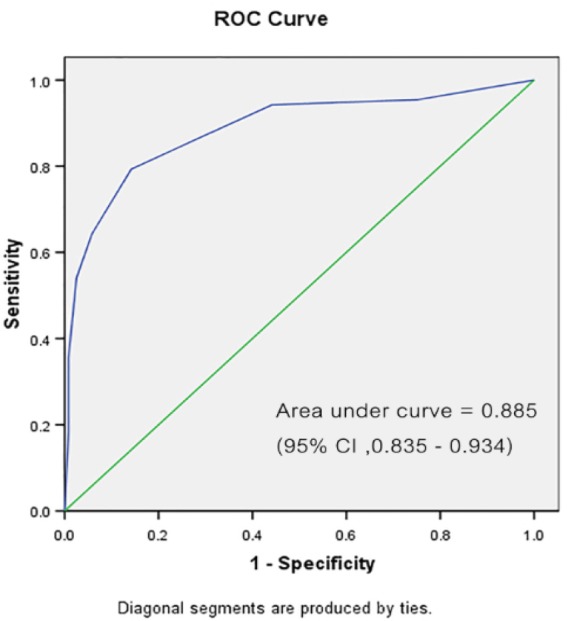
Receiver Operator Characteristic Curve for Modified Reid Colposcopic Index. The green line is the line of equality. (CI, confidence interval)

During the study period, 210 women were initially identified. Of those, 3 women were excluded due to inadequate biopsy specimens. Therefore, 207 women were eligible. All patients were Asian. The average age was 35.48 ±10.63 years (range 17-85 years). Of those, 188 women (90.8%) aged less than 50 years. Forty-eight women (23.2%) were nulliparous. Anti-HIV tests were positive in 13 women (6.3%).Regarding referral cytology, the rate of ASC-US, LSIL, HSIL and positive for malignancy cytology were 16.9%, 37.2%,31.0% and 3.9%, respectively. In colposcopy, satisfactory colposcopy (T-zone type 1 and 2) was noted in 138 (66.7%) women. Most of colposcopic scoring were recorded by using only the first three criteria (abbreviated RCI) as the iodine staining was done in only 58 of 207 women (28.01%). Large lesions (3-4 quadrants) were noted in 58 (28%) and deep endocervical involvement in which the upper border of lesion cannot be seen was observed in 29(28.5%). Table 2 illustrates the demographic data. There was no woman with complication from CDB. Of the 207 CDB histology samples, 58.0% were CIN1 or less, 42.0% were CIN2 or worse. One case of invasive cancer was found from CDB. The CDB histology was categorized in to two group ; “CIN1 or less” and “CIN2 or worse”. After collecting more parameter from charts of 207women, the new scoring system was calculated. The performance measured by sensitivity and specificity PPV and NPV is clearly demonstrated (Table 3). As shown, a score of 6 or more has a high specificity of 97.5% (95% CI = 94.7-100.2) and positive predictive value of 94.0% (95%CI = 87.4-100.5) for predicting biopsy-proved CIN2 or worse with a sensitivity of 54 %(95%CI =43.5-64.5). Lowering the score to 4 or more can improve the sensitivity to 79.3% (95%CI = 70.8-87.8) while decreasing the specificity to 85.8 (95%CI = 79.5-92.0) for predicting CIN2 or worse. Lower scores also shows high negative predictive values; a score of 2 or more resulted in a negative predictive value of 93% (95%CI = 87.1-98.9) and high sensitivity at 94.2 % (95%CI = 89.3-99.1). Figure 1 shows the area under ROC curve of 0.885 (95%CI =0.835-0.934) which shows a good diagnostic test.

**Figure 2:**
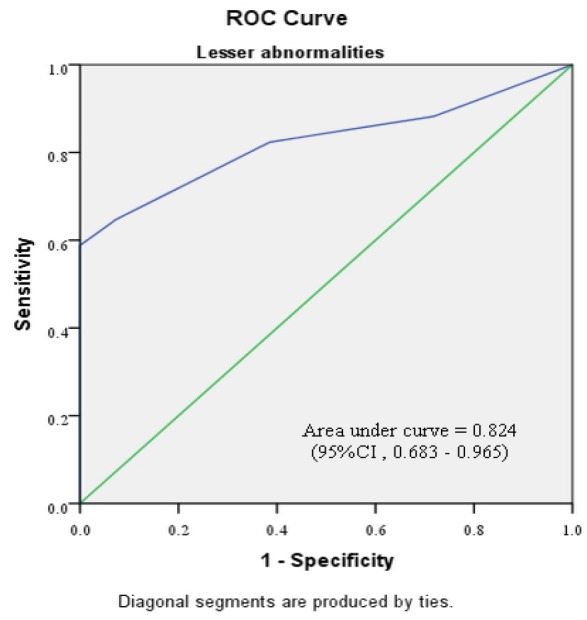
Receiver Operator Characteristic Curve for Modified Reid Colposcopic Index in Cases Preceded by “Lesser Abnormalities”. The green line is the line of equality. (CI, confidence interval)

**Figure 3:**
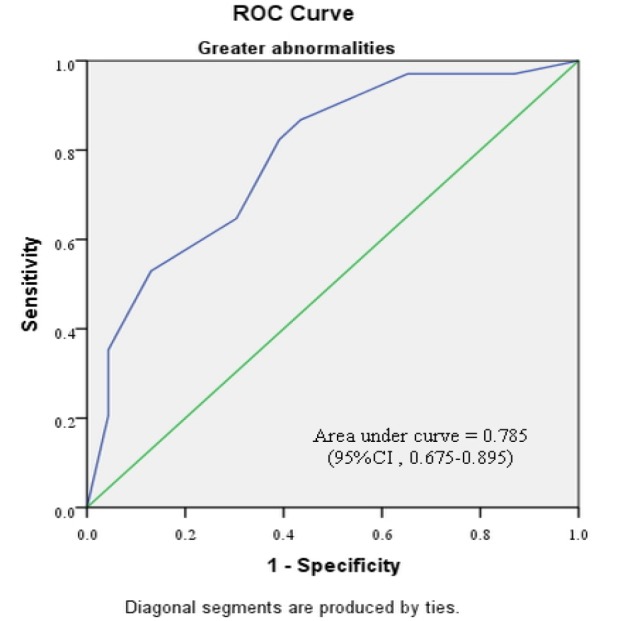
Receiver Operator Characteristic Curve for Modified Reid Colposcopic index in Cases Preceded by “Greater Abnormalities”. The green line is the line of equality. (CI, confidence interval)

**Figure 4:**
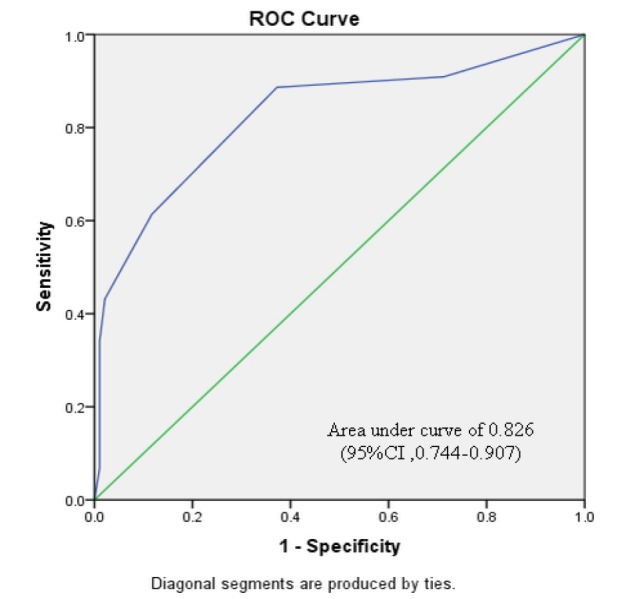
Receiver Operator Characteristic Curve for Modified Reid Colposcopic Index in Cases with Satisfactory Colposcopy (type 1,2 T-zone). The green line is the line of equality. (CI, confidence interval)

**Figure 5:**
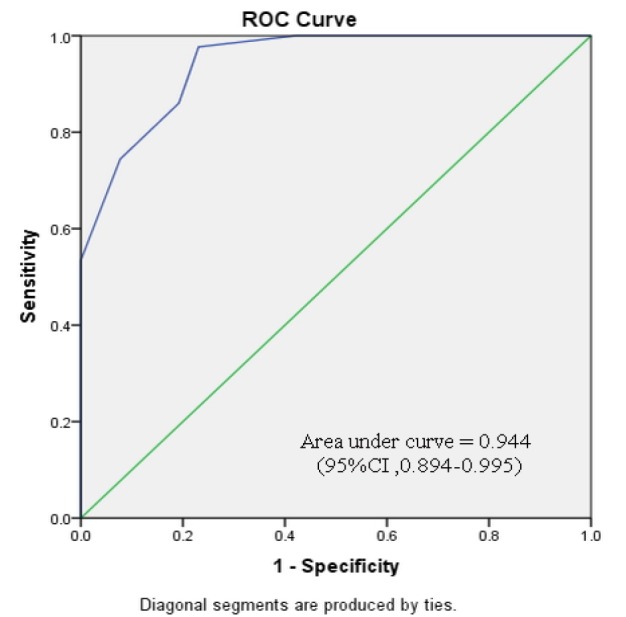
Receiver Operator Characteristic Curve for Modified Reid Colposcopic Index in Cases with Unsatisfactory Colposcopy (Type3 T-zone). The green line is the line of equality. (CI, confidence interval)

Subgroup analyses were performed, based on the severity of preceding cervical cytology and type of cervical transformation zone visualization (Table 4-7). Figure 2-5 shows the performance of our modified RCI in subpopulation. This scoring system had greater discriminatory ability in a group with type 3 T- zone (Table 7). The area under ROC curve was 0.944 (95%CI =0.894-0.995) which was excellent (Figure 5).

## Discussion

The colposcopy plays a major role in the management of women with abnormal cervical screening test. Women with high-grade lesion are offered treatment, and those with lesser abnormalities can be followed at colposopy clinic or returned to routine screening (Massad et al., 2013). Inaccurate colposcopy can result in a delay in diagnosis and treatment, unnecessary repeated colposcopy, cytology and treatment. Interpretation of colposcopic findings is subjective as change in findings of aceto-whiteness or vessel appearance is not highly specific to CIN. Squamous metaplasia, repair and regeneration, inflammation, and infection may all produce abnormal colposcopic transformation zone findings, such as acetowhite epithelium and abnormal vessels (Sellors and Sankaranarayanan, 2003; Campion and Canfell, 2015). There has been more concern regarding of the value of well-known RCI. Despite widespread use, some studies reported imperfect accuracy and correlation of RCI (Ferris and Litaker, 2006; Massad et al., 2009). Colposcopists using the abbreviated RCI in ALTS (Atypical Squamous Cells of Undetermined Significance and Low-grade Squamous Intraepithelial Lesion Triage Study) failed to detect CIN2 or higher at levels expected (Ferris and Litaker, 2006).The sensitivity for CIN2+ (CIN2 or worse) of a high grade diagnosis by RCI was only 30% in one study (Massad et al., 2009). A multivariate analysis by Shaw et al., (2003) found that, after evaluating the individual variables within the RCI, only the degree of acetowhite change was able to significantly predict CIN. 

In addition, some emerging or important colposcopic signs such as “ridge sign” or cuffed crypt openings which are hallmark of high-graded lesion are not included in RCI (Sellors and Sankaranarayanan, 2003; Scheungraber at al., 2009). It is well documented that the size of a lesion which is not included in RCI does correspond to the severity of CIN and has a strong prediction of high-grade lesion or occult invasive. From the study conducted by Strander et al. (2005) , size as a criterion contributed more to the achievement of Swede scoring system than the more widely used criteria of “vessel patterns” and “iodine uptake”. Theoretically, large low-grade lesions may harbor foci of evolving high-grade disease and that size of a low-grade lesion may be a predictor of risk of undetected high-grade disease. The addition of size of lesion as an independent predictor of disease severity is likely to be of value (Campion and Canfell, 2015). The location of the lesion relative to the squamocolumnar junction is also associated with severity of lesion or the presence of occult invasive disease (Sellors and Sankaranarayanan, 2003; Campion and Canfell, 2015). Hong et al., (2010) reported the good performance of their modified RCI that omitted the iodine staining procedure and replaced with the location of the lesion in the transformation zone. The authors’ proposed colposcopic index has major advantage that our scoring incorporates lesion size and location of lesion relative to endocervical canal as an independent criterion. Our colposcopic index is adapted from the RCI in combination with Swede score (Strander et al., 2005). In our database, the details of size and location of the lesions were not available in all cases. Data can be obtained from either existing document or retrospective reviewing of digitized colpophotograpy. Also, endocervical undefined lesion is scored as 2 points in this criterion. This is based on the common knowledge that the areas of high-grade lesion are frequently evolving in the proximal edge of the existing low-grade lesion. With extensive squamous metaplasia from aging, the squamocolumnar junction moves cephalad into the endocervical canal. Therefore, the lesion with invisible upper border predicts the presence of high-grade lesion (Sellors and Sankaranarayanan, 2003; Campion and Canfell, 2015).

**Table 1:** The Author’s Proposed “Modified Reid Colposcopicindex

Colposcopic signs	0	1	2
Margin	Condylomatous, Micropapillary, Indistinct aceto-white, Flocculated, featured, Angular, jagged, Satellite lesion	Regular lesions, Smooth, straight outline	Rolled, peeling edge, Internal demarcations between areas
Color	Shinny, snow-white, Indistinct aceto-white	Intermediate (shinny gray)	Dull, oyster-white
Vessels	Fine-caliber, poorly formed patterned, condylomatous or micropapillary	Absent	Definite punctation or mosaic
Size	< 5 mm or < 1 quadrant	5-15 mm or 2 quadrants	>15 mm or 3-4 quadrants or endocervically undefined

**Table 2:** Demographic and Colposcopy Data for Women Recruited

Demographic	
Age :mean±SD (years)	35.48 ±10.63
Age range (years)	17-85
Ethnicity	Asian
Thai	206 (99.5)
Burmeses	1 (0.5)
Province	
Bangkok	112 (54.1)
Other	95 (45.9)
Smoking	
Current	9 (4.3)
Ex-smoker	13 (6.3)
Never	81 (39.1)
Unknown	72 (34.8)
Parity	1.53±1.35
0	48 (23.2)
1	66 (31.9)
2	56 (27.1)
3	23 (11.1)
4	7 (3.4)
≥5	7 (3.4)
Anti-HIV positive	13 (6.3)
Referral cytology	Conventional method
Lesser abnormalities	
NILM	1 (0.5)
ASC-US	35 (16.9)
LSIL	77 (37.2)
Greater abnormalities	
ASC-H	17 (8.2)
HSIL	64 (31)
AGC-NOS,AGC-FN	1 (0.5),1 (0.5)
Invasive carcinoma	8 (3.9)
N/A	3 (1.4)
Cytology-colposcopy interval: mean±SD (day)	64.9 ±53.3
Malignancy cytology positive	8 (3.9)
Colposcopy	
Satisfactory(type1,2)	138 (66.7 )
Large (3-4 quardrants) lesion	25 (28)
Lesion extending into endocervical canal	59 (28.5)
Subjective colposcopy impression	
Normal	3 (1.4)
LSIL	121 (58.4)
HSIL	78 (37.7)
Invasive	3 (1.4)
Other	2 (1)
Colposcopically directed biopsy (CDB)	
Negative	25 (12.1)
LSIL	95 (45.9)
HSIL	86 (41.5)
Invasive	1 (0.5)

**Table 3:** Performance of Modified Reid Colposcopic Index

Score	Sensitivity, %	Specificity, %	PPV, %	NPV, %	Accuracy, %
	(95% CI)	(95% CI)	(95% CI)	(95% CI)	(95% CI)
≥ 0	100	0	42.0 (35.3-48.7)	N/A	42.0 (35.3-48.7)
≥ 1	95.4 (91.0-99.8)	25.0 (17.2-32.7)	47.9 (40.5-55.4)	88.2 (77.4-99.0)	54.5 (47.8-61.3)
≥ 2	94.2 (89.3-99.1)	55.8 (46.9-64.7)	60.7 (52.5-68.9)	93.0 (87.1-98.9)	71.9 (65.8-78.1)
≥ 3	83.9 (76.1-91.6)	76.6 (69.1-84.2)	72.2 (63.5-81.0)	86.7 (80.3-93.2)	79.7 (74.2-85.1)
≥ 4	79.3 (70.8-87.8)	85.8 (79.5-92.0)	80.2 (71.8-88.6)	85.1 (78.7-91.4)	83.0 (77.9-88.2)
≥ 5	64.3 (54.3-74.4)	94.1 (89.9-98.3)	88.8 (81.1-96.6)	78.5 (71.7-85.1)	81.69 (76.3-86.9)
≥ 6	54.0 (43.5-64.5)	97.5 (94.7-100.2)	94.0 (87.4-100.5)	74.5 (67.7-81.3)	79.2 (73.7-84.7)
≥ 7	35.6 (25.5-45.7)	99.1 (97.5-100.7)	96.8 (90.8-102.9)	68.0 (61.0-74.9)	72.4 (66.3-78.5)
8	18.3 (10.2-26.5)	99.1 (97.5-100.7)	94.1 (82.9-105.3)	62.6 (55.7-69.5)	65.2 (58.7-71)

**Table 4:** Performance of Modified Reid Colposcopic Index in Cases Preceded by “Lesser Abnormalities”

Score	Sensitivity, %	Specificity, %	PPV, %	NPV, %	Accuracy, %
	(95% CI)	(95% CI)	(95% CI)	(95% CI)	(95% CI)
≥ 0	100	0	15.0 (8.4-21.6)	N/A	15.0 (8.4-21.6)
≥ 1	88.2 (72.9-103.5)	28.1 (19.1-37.1)	17.8 (9.6-26.0)	93.1 (83.8-102.3)	37.1 (28.2-46.0)
≥ 2	82.3 (64.2-100.4)	61.4 (51.7-71.1)	27.4 (15.2-39.7)	95.1 (89.8-100.5)	64.6 (55.7-73.4)
≥ 3	70.5 (48.9-92.2)	82.2 (74.6-89.9)	41.3 (23.4-59.3)	94.0 (88.9-99.1)	80.5 (73.2-87.8)
≥ 4	64.7 (41.9-87.4)	92.7 (87.5-97.9)	61.1 (38.5-83.6)	93.6 (88.7-98.5)	88.5 (82.6-94.3)
≥ 5	58.8 (35.4-82.2)	100	100	93.2 (88.3-98.0)	93.8 (89.3-98.2)
≥ 6	52.9 (29.2-76.6)	100	100	92.3 (87.1-97.4)	92.9 (88.1-97.6)
≥ 7	29.4 (7.7-51.0)	100	100	88.8 (82.9-94.8)	89.3 (83.7-95.0)
8	5.8 (-5.3-17.0)	100	100	85.7 (79.2-92.2)	85.8 (79.4-92.2)

Lesser abnormalities include; NIL, ASC-US or LSIL cytology

**Table 5:** Performance of Modified Reid Colposcopic Index in Cases Preceded by “Greater Abnormalities”

Score	Sensitivity, %	Specificity, %	PPV, %	NPV, %	Accuracy, %
	(95% CI)	(95% CI)	(95% CI)	(95% CI)	(95% CI)
≥ 0	100	0	74.7 (65.8-83.6)	N/A	74.7 (65.8-83.6)
≥ 1	97.0 (93.0-101.0)	13.0 (-0.7-26.8)	76.7 (67.8-85.6)	60.0 (17.0-102.9)	75.8 (67.0-84.6)
≥ 2	97.0 (93.0-101.0)	34.7 (15.3-54.2)	81.4 (73.0-89.9)	80.0 (55.2-104.7)	81.3 (73.3-89.3)
≥ 3	86.7 (78.7-94.8)	56.5 (36.2-76.7)	85.5 (77.2-93.8)	59.0 (38.5-79.6)	79.1 (70.7-87.4)
≥ 4	82.3 (73.2-91.4)	60.8 (40.9-80.8)	86.1 (77.7-94.5)	53.8 (34.6-73.0)	76.9 (68.2-85.5)
≥ 5	64.7 (53.3-76.0)	69.5 (50.7-88.3)	86.2 (76.8-95.7)	40.0 (24.8-55.1)	65.9 (56.2-75.6)
≥ 6	52.9 (41.0-64.8)	86.9 (73.1-100.7)	92.3 (83.9-100.6)	38.4 (25.2-51.6)	61.5 (51.5-71.5)
≥ 7	35.2 (23.9-46.6)	95.6 (87.3-103.9)	96.0 (88.3-103.6)	33.3 (21.9-44.7)	50.5 (40.2-60.8)
8	20.5 (10.9-30.2)	95.6 (87.3-103.9)	93.3 (80.7-105.9)	28.9 (18.7-39.1)	39.5 (29.5-49.6)

Greater abnormalities include; ASC-H , HSIL, AGC, AIS, invasive carcinoma

**Table 6:** Performance of Modified Reid Colposcopic Index in Cases with Satisfactory Colposcopy (Type 1, 2 T-zone)

Score	Sensitivity, %	Specificity, %	PPV, %	NPV, %	Accuracy, %
	(95% CI)	(95% CI)	(95% CI)	(95% CI)	(95% CI)
≥ 0	100	0	31.8 (24.1-39.6)	N/A	31.8 (24.1-39.6)
≥ 1	90.9 (82.4-99.4)	28.7 (19.5-37.8)	37.3 (28.2-46.5)	87.1 (75.3-98.9)	48.5 (40.2-56.8)
≥ 2	88.6 (79.2-98.0)	62.7 (52.9-72.5)	52.7 (41.3-64.0)	92.1 (85.6-98.7)	71.0 (63.4-78.5)
≥ 3	68.1 (54.4-91.9)	81.9 (74.1-89.7)	63.8 (50.0-77.5)	84.2 (77.2-92.0)	77.5 (70.5-84.5)
≥ 4	61.3 (46.9-75.7)	88.3 (81.8-94.8)	71.0 (56.6-85.4)	83.0 (75.6-90.3)	79.7 (73.0-86.4)
≥ 5	43.1 (28.5-57.8)	97.8 (94.9-100.7)	90.4 (77.9-103.0)	78.6 (71.2-86.0)	80.4 (73.8-87.0)
≥ 6	34.0 (20.0-48.1)	98.9 (96.8-101.0)	93.7 (81.8-105.6)	76.2 (68.6-83.7)	78.2 (71.3-85.1)
≥ 7	18.1 (6.7-29.5)	98.9 (96.8-101.0)	88.8 (68.3-109.4)	72.0 (64.3-79.8)	73.1 (65.8-80.5)
8	6.8 (-0.6-14.2)	98.9 (96.8-101.0)	75.0 (32.5-117.4)	69.4 (61.6-77.2)	69.5 (61.8-77.2)

**Table 7:** Performance of Modified Reid Colposcopic Index in Cases with Unsatisfactory Colposcopy (Type 3 T-zone)

Score	Sensitivity, %	Specificity, %	PPV, %	NPV, %	Accuracy, %
	(95% CI)	(95% CI)	(95% CI)	(95% CI)	(95% CI)
≥ 0	100	0	62.3 (50.8-73.7)	N/A	62.3 (50.8-73.7)
≥ 1	100	11.5 (-0.7-23.8)	65.1 (53.6-76.6)	100	66.6 (55.5-77.7)
≥ 2	100	30.7 (13.0-48.5)	70.4 (59.0-81.9)	100	73.9 (63.5-84.2)
≥ 3	100	57.6 (38.7-76.6)	79.6 (68.8-90.3)	100	84.0 (75.4-92.7)
≥ 4	97.6 (93.1-102.1)	76.9 (60.7-93.1)	87.5 (78.1-96.8)	95.2 (86.1-104.3)	89.8 (82.7-96.9)
≥ 5	86.0 (75.6-96.4)	80.7 (65.6-95.9)	88.1 (78.3-97.8)	77.7 (62.1-93.4)	84.0 (75.4-92.7)
≥ 6	74.4 (61.3-87.4)	92.3 (82.0-102.5)	94.1 (86.2-102.0)	68.5 (53.1-83.9)	81.1 (71.9-90.3)
≥ 7	53.4 (38.5-68.4)	100	100	56.5 (42.2-70.8)	71.0 (60.3-81.7)
8	30.2 (16.5-43.9)	100	100	46.4 (33.3-59.4)	56.5 (44.8-68.2)

Iodine staining is not routinely performed in practice because of its inconvenience and poor predictive value of high-grade lesion (Reid and Scalzi, 1985; Boonlikit, 2011; Campion and Canfell, 2015). For this reason, we replace this criterion with size and location of lesion as the total score is still equal to 8 which is resemble to RCI and familiar to practicing colposcopists. The diagnostic performance of our test was evaluated using ROC curve analysis and the area under curve (AUC) of 0.87 showed good accuracy to discriminate diseased cases from normal cases. The outcome is comparable with our previous work from the same institute (Boonlikit, 2016). In the present study, diagnostic accuracy and performance of this modified RCI varied, depending on various factors such as the type of preceded cervical cytology and completeness of transformation zone visualization. In women preceded by “lesser abnormalities”(NIL, ASC-US or LSIL), our colposcopic index had a lower sensitivity and higher specificity as compared to women preceded by “greater abnormalities”(ASC-H, HSIL, AGC, AIS, invasive carcinoma) or in women with all degrees of abnormal cytology. This is consistent with previous studies focusing on women with preceding ASCUS or LSIL cytology which reported low sensitivity and high specificity (Ferris and Litaker, 2006; Massad et al., 2009). In studies performed in an unselected population with all degrees of abnormal cytological results, higher sensitivity and specificity were observed (Mousavi et al., 2007; Boonlikit, 2016). A possible explanation is that the limitation of colposcopy and directed biopsy sampling is their inability to detect small CIN2 and CIN3 lesions (Jeronimo and Schiffman, 2006). In women with “greater abnormalities”, they are more likely to have histology-proven CIN 2/3 or cancer and tend to have large, obvious lesions that are likely to be detected by colposcopy. Interestingly, the performance of our modified RCI was very excellent in group with incomplete transformation zone visualization (type 3 T-zone). This could be explained by the fact that the proportion of “greater abnormalities” was higher in this group as compared to satisfactory colposcopy group (65% vs. 33%) resulting in high performance of our colposcopic index.

In addition, the result showed that increasing score cut-off decreased the sensitivity and increased the specificity. Changing the cut-off level significantly affects the performance of colposcopic index. In general, the optimal cutoff value for our colposcopic index to define histology-proven CIN2+ with satisfactory sensitivity and specificity is 3. However, it is possible to select various cut-off values according to the clinical aim. Based on this concept, Swede score has been widely used as the lower threshold (5/10) with high sensitivity can be used for screening, whereas the higher threshold (8/10) with high specificity can be used for screen-and-treat selection to decrease the overtreatment rate (Ranga et al., 2017). From the present study, the proposed scoring system has 2 suitable cut off values. The use of a high cut-off (≥6/8) which has increased specificity at time of colposcopy could be helpful when triaging women through colposcopy, and when treatment at first visit is being considered. Currently, see-and-treat LLETZ is acceptable option for women with HSIL cytology (Massad et al., 2013). It is based exclusively on colposcopy assessment at initial visit. The disadvantage is the risk of having the negative histology which is considered as over-treatment (Greenberg, 2002). Histologic study of loop-excised specimens removed at a “see-and-treat” approach revealed no disease in 5% to 40% of specimens (Campion and Canfell, 2015). As a result, women are put at risk of unnecessary morbidity, which incurs extra costs to the health service. Using high-specificity colposcopics score could be helpful in this setting. High cut-off in our scoring system can reduce risk of over-treatment in this regard. The specificity for a total score of 6 or more has a high specificity of 97.5% and positive predictive value of 94.0% for predicting biopsy-proved CIN2 or worse which implies that at this level the patient can be managed by ‘see-and-treat’ with a decreasing rate of overtreatment. Conversely, using low cut-off value(≥ 2/8) in our scoring system may safely omit CDB in majority of cases. The high negative predictive value of 93.0% and high sensitivity at 94.2% for cut-off value ≥ 2/8 indicate that patients with total score below this cut-off can be followed safely without the need for CDB which is very useful in the situation where the risk of taking biopsy is high (i.e. pregnancy, patients with coagulation disorder). 

Certainly, limitations of the present study do exist. First, its retrospective nature precludes standardization of protocol. Second ,the study design is not representative of the performance of “real-time” clinical colposcopic score as retrospective incorporating additional criterion to existing RCI in the database for the purpose of recalculating new colposcopic scoring may be inappropriate. In addition, since the iodine staining is not routinely applied in our practice, performance of traditional RCI and our colposcopic index cannot be compared. In the present study, small histologic sample from CDB might not be accurate endpoint for diagnosis of the SIL. Further excisional treatment occasionally revealed different histologic diagnosis. Finally, the lack of histology review of all CDB may be considered a limitation of the present study.

In conclusion, the authors’ proposed score was evaluated. It simply develops from RCI which is the most well-known scoring system. Replacing iodine staining with the size and location of the lesion renders the colposcopic score practical and beneficial. High cut-off level can be used in see-treat management, enabling the single-visit approach with confidence. Low cut-off level might be used for omitting biopsy in case of low grade impression. This is particularly useful in the situation where the risk from taking biopsy outweighs the benefit. This scoring system seems to have greater performance in women with incomplete transformation zone visualization.
